# Comparison of multiple international metrics for benchmarking antibiotic usage (ABU) using UK beef and sheep data

**DOI:** 10.1093/jac/dkad259

**Published:** 2023-08-16

**Authors:** Peers Davies, Robert Hyde, Alexander Corbishley

**Affiliations:** Department of Livestock and One Health, Institute of Infection, Veterinary and Ecological Sciences, University of Liverpool, Leahurst Campus, Wirral, UK; School of Veterinary Medicine and Science, University of Nottingham, Sutton Bonington Campus, Leicestershire, UK; Royal (Dick) School of Veterinary Studies and The Roslin Institute, University of Edinburgh, Easter Bush Campus, Midlothian, UK

## Abstract

**Background:**

Accurate surveillance of livestock antibiotic usage (ABU) at the farm level is an increasingly important part of national antibiotic stewardship initiatives. Numerous ABU indicators or metrics have been developed in Europe and North America but the comparability of these metrics is poorly understood. For policymakers, understanding the relationship between metrics is important when considering the risks posed by ABU and how to regulate them, at the national level, and regulate international trade access in livestock products between countries who use different ABU metrics.

**Objectives:**

To quantify the patterns of ABU among beef (cattle) and lamb (sheep) production systems. To explore ABU variation between farm types across seven ABU metrics developed in Europe and North America using a common dataset of sheep and beef farms’ antibiotic purchases from the UK.

**Methods:**

A dataset of >16 200 antibiotic sales events to 686 farm enterprises of different types underwent quantitative analysis. Correlation matrixes were generated for seven international ABU metrics.

**Results:**

ABU was significantly higher among calf-rearers. Across all farm types, tetracyclines and β-lactams were the predominant groups by mass, but represent a similar dose equivalent to macrolides across most farm types. Good agreement (>0.9) was observed between metrics.

**Conclusions:**

Reliable metrics to accurately benchmark farms are crucial for maintaining confidence of farmers in the fairness of any surveillance system, especially when the ranking of any given system may be linked to financial subsidies or penalties and also when negotiating import and export access for livestock products between countries.

## Introduction

Antibiotic resistance (ABR) has previously been linked to antibiotic usage (ABU) at a national level.^1^ The relationship between antibiotic use and resistance is complex because ABR is multifactorial, with many potential non-antibiotic causal factors^2,3^ as well as antibiotic use factors that influence the emergence, abundance, diversity and dissemination of ABR. Veterinary use of antibiotics has been a policy and research focus for over a decade, with an emphasis upon monogastric species because of the very high ABU in the pig and poultry sectors, associated with intensive production systems. Ruminant livestock farming, specifically beef cattle and sheep farming systems, differ from these livestock sectors in a number of important respects. Firstly, beef and sheep farming systems are more extensive than the other livestock sectors, utilizing 73% of grazing land in the UK, for example.^4^ Secondly, beef and sheep farmers represent the majority (69%) of active farmers in the UK, but are also the most economically marginal farming enterprises, making them particularly vulnerable to a wide range of external challenges. When policymakers consider appropriate regulatory instruments to control ABU that may place additional costly or labour-intensive obligations upon all livestock producers it is essential that the impact on beef and sheep farmers is considered. Overly simplistic policies may burden a very large number of marginal businesses with additional bureaucratic tasks, costs and restrictions that are not proportionate to the actual ABR risk that their farming systems pose. To better understand the relative contribution of different livestock sectors to the overall pattern of veterinary ABU we need granular data that describe the distribution of ABU across farms operating comparable production systems. The aim of this study was to quantitatively describe for the first time the patterns of ABU across all the different main classifications of beef, sheep and mixed (beef and sheep) farms by all the available and applicable national and international ABU metrics, in order to assess the level of agreement between these metrics.

In the UK, antibiotic supply to farms is only legally permitted by prescription from a veterinary surgeon with active responsibility for the animals on that farm. The vast majority of these antibiotic prescriptions are fulfilled by the issuing veterinary practice, with the remainder supplied by a third-party pharmacy according to the prescription provided by the veterinary practice. This regulatory structure provided a data collection opportunity where electronic sales/prescription data can be collated from the veterinary practice for analysis and benchmarking. This methodology has been used successfully in cross-sectional and longitudinal ABU studies in sheep and dairy.^5–7^ To our knowledge, this is the first study to apply the methodology to a large dataset of beef cattle and mixed sheep/beef enterprises from multiple independent veterinary practices.

Monitoring ABU in mixed cattle and sheep farms is challenging when using the conventional European Surveillance of Veterinary Antibiotic Consumption (ESVAC)^8^ metrics as these metrics were originally designed for national rather than farm-level benchmarking. Beef ABU is particularly challenging due to the ESVAC metric requirement for monitoring the number of slaughtered animals, as not all beef farms produce animals for slaughter. Furthermore, the breeding cow is excluded from the population correction unit (PCU), as defined by ESVAC. These factors, combined with the longevity of suckler cows, can inflate the apparent ABU on beef suckler farms when comparisons are made with other farming systems using metrics based on the ESVAC mg/kg PCU. More recently, the use of UK metrics avoiding the need to collect the number of slaughtered animals has been proposed.^9^ These metrics incorporate standardized weights for multiple age ranges, thereby allowing a denominator biomass to be calculated for a wider range of farm types that buy and sell animals at various ages instead of, or in addition to, selling animals direct to slaughter.

Whilst there are a variety of metrics available for the calculation of ABU, examples of comparisons between ABU metrics are rare. Previous studies have examined correlations between ABU metrics for dairy cattle,^10^ pigs^11^ and beef feedlots^12^ but not examined the more complex relationships between metrics in mixed species farm systems as we have addressed here. There is also a need to understand how comparable ABU metrics are between countries when policymakers are considering the equivalence of ABU standards. The equivalence of ABU metrics and benchmarking systems between countries is an essential element in evaluating ABR risk of food products when negotiating trade access. This is particularly important for counties that export a significant proportion of their livestock produce into markets that use different ABU metrics. The UK and EU are good examples of this type of trade relationship; the UK exports a large proportion of its lamb meat to the EU while importing from the EU a significant proportion of the beef that is consumed in the UK. Many large-scale red meat exporting countries in Asia, Africa and South America do not currently operate any form of ABU benchmarking. In order to gain or maintain access to high-value export markets, such as the EU, the adoption of a suitable ABU metric and surveillance system may be necessary for these exporting nations. In this study, agreement between seven metrics from five different national and international ABU surveillance regimens are compared (Canada, UK, Netherlands, Denmark and EU) for two livestock species (sheep and beef cattle) using a large dataset of farms across seven production system categories.

## Materials and methods

The study was conducted under University of Edinburgh (HERC_141_17) ethical approval. All UK veterinary practices registered with the Royal College of Veterinary Surgeons (RCVS) who self-declared as treating cattle and sheep were invited by e-mail to participate in the study (*n* = 568). Anonymized antibiotic product prescription and sales records were collated from 30 veterinary practices that were able to contribute sales and prescription records for all antibiotic products supplied to a minimum of 10 sheep and/or beef cattle commercial farm enterprises per practice. Practices were recruited with client farms located in the following regions: North Wales, West Wales, Mid Wales, South Wales, Central Scotland and the Scottish Borders, and the following English regions: South West, South East, West Midlands, East Midlands, North East, North West and East Anglia. Each practice provided details of all antibiotic products prescribed and/or supplied to all their clients during the study period including data, product name and quantity. Anonymized farm metadata on production system demographics and management practices were collected for each farm by questionnaire (see Appendix iii, available as Supplementary data at *JAC* Online) and linked to antibiotic supply data by a unique identifier coded by each veterinary practice. A total of 16 208 antibiotic sale events of 1221 unique products recorded within the database from a consecutive 12 month period were recorded from 686 beef cattle farms, sheep farms and mixed sheep and beef cattle farms. For the mixed (beef and sheep) farms, ABU was not hypothecated by species. Beef cattle farms were further classified by management system into either suckler (adult breeding females more than 25% of herd), grower/finisher (majority of youngstock >12 months old and not a suckler herd) and calf-rearer (majority youngstock <12 months old and not a suckler herd). Numbers of each category are detailed in Table 1.

**Table 1.** Summary of mean, median and IQR of ABU in 686 sheep and beef farms by a variety of metrics

Metric	Parameter	Sheep and calf-rearer	Sheep and grower-finisher	Sheep and suckler	Calf-rearer	Grower-finisher	Suckler	Sheep
mg/PCU (EU)	Mean	12.81	10.79	7.92	19.51	8.02	12.47	10.67
	Median	5.20	6.62	4.00	12.82	4.32	3.91	6.39
	IQR	14.4	13.91	8.34	25.20	10.31	8.85	10.45
DDDvet (EU)	Mean	0.99	1.25	2.13	2.09	1.00	2.01	1.46
	Median	0.46	0.71	0.60	1.47	0.46	0.42	0.71
	IQR	1.54	1.54	1.49	2.55	1.32	1.71	1.40
DCDvet (EU)	Mean	0.30	0.35	0.63	0.56	0.28	0.44	0.36
	Median	0.11	0.19	0.17	0.43	0.13	0.12	0.19
	IQR	0.43	0.43	0.55	0.65	0.37	0.49	0.35
mg/kg (UK)	Mean	8.56	8.33	4.93	11.92	5.65	5.02	8.47
	Median	4.84	5.48	2.90	7.73	2.90	1.41	4.49
	IQR	12.24	10.42	5.20	14.43	7.32	2.78	7.61
DDD (NLD)	Mean	2.00	2.17	1.39	0.95	0.62	0.77	—
	Median	0.88	1.22	0.58	0.51	0.29	0.17	—
	IQR	2.51	2.54	1.32	1.36	0.81	0.48	—
DAPD (DAN)	Mean	4.31	5.30	2.91	7.59	3.88	3.68	4.93
	Median	2.37	2.98	1.28	6.51	2.01	1.35	1.81
	IQR	5.28	5.36	2.16	8.78	5.00	3.54	3.81
mg/PCU (CAN)	Mean	27.22	29.15	14.10	6.85	7.46	7.54	—
	Median	9.56	15.95	5.77	4.88	3.91	2.42	—
	IQR	24.69	34.03	14.33	8.70	9.45	4.60	—

Metrics: mg/PCU (ESVAC, 2020); DDDvet (ESVAC, 2020); DCDvet (ESVAC, 2020); mg/kg (UK) (CHAWG, 2020); DDD (The Netherlands; NLD) (SDa, 2019); DAPD (Denmark: DAN) (DANMAP, 2019); mg/PCU (Canada: CAN) (CIPARS, 2015),^8,9,13–15^ Data are partitioned by farm system type.

As previous studies have shown that metrics correlate relatively well for pig farms,^11^ a similar approach was adopted for the beef and sheep farm data, with analysis of a variety of metrics used globally to test for correlation. The following metrics were included for analysis: mg/PCU (EU) (ESVAC, 2020); DDDvet (EU) (ESVAC, 2020); DCDvet (EU) (ESVAC, 2020); mg/kg (UK) (CHAWG, 2020); DDD (The Netherlands) (SDa, 2019); DAPD (Denmark) (DANMAP, 2019); and mg/PCU (Canada) (CIPARS, 2015).

The geographical origin of each metric is supplied in parentheses. References for the full methodology of each metric and a summary of numerator and denominator populations are included in Appendix i.

### Numerator calculation

The fuzzyjoin R package^16^ was used to pair Veterinary Medicines Directorate (VMD)-registered product names with practice-recorded product names. This was then checked manually to ensure correct pairing of products. Products that were not listed in the VMD cattle/sheep list (e.g. Linco-Sol powder) were entered manually. Aerosol products were checked manually, as some were recorded on a volume basis (i.e. 422 or 211 mL), with others were recorded as a single unit.

The calculation of dose-based metrics was based on standardized dose information for DDDvet, DCDvet, DDD (The Netherlands) and DAPD (Denmark), respectively (Appendix i).^13^ DAPD was calculated per 1000 animal days. Products were paired by their EU Anatomical Therapeutic Chemical classification system code for veterinary medicinal products (ATCvet code) where available, with dosages for unpaired products being calculated as the mean dosage for that particular antibiotic class. The calculation of mass-based metrics was based on the milligrams of antibiotic used (factor corrected for procaine benzylpenicillin and penethemate) divided by the denominator population, as described below.

### Denominator calculation

As lamb numbers were unavailable for the majority of farms, lamb numbers have been estimated by multiplying the number of ewes by the Agriculture and Horticulture Development Board (AHDB) published average rearing percentage (143.5%) according to the methodology previously described by Davies *et al*.^5^ Ewe and estimated lamb numbers were used to calculate PCU by standard ESVAC methodology.

For beef farms, slaughter statistics were not always directly available for the calculation of the PCU. An estimation of slaughtered animals was derived from the total number of cattle reported as sold ‘finished’ and cattle recorded as sold over 1 year old.

Animals recorded as breeding cows were assumed to be a beef-sired female, with all other animals using aggregated weight across AHDB breed type, sex and age group if unknown (i.e. mean female and male weights for a given age group were used if unknown). Canadian denominator populations were calculated from the Canadian integrated programme of antibiotic resistance and surveillance.^14^

Farm management system was created based on the CHAWG antimicrobial usage (AMU) benchmarking guidelines^9^ for bovines, into either suckler (adult breeding females more than 25% of herd), grower/finisher (majority of youngstock >12 months old and not a suckler herd) and calf-rearer (majority youngstock <12 months old and not a suckler herd).

The metrics DDD (The Netherlands) and mg/PCU (Canada) should be interpreted with caution for sheep and mixed farms as they do not include a sheep denominator and therefore use all of the farms’ numerator doses with only part of their denominator mass.

### Statistical analysis

Statistical analysis was performed using R statistical software.^17^ Data were filtered to exclude variables where >20% of observations contained missing variables. Random forest imputation was then used to impute missing values using the rfImpute function within the randomForest package.^18^ Correlations between ABU metrics were analysed using Spearman’s rank (i.e. the relationship between the farm-year ABU rank between different metrics). Univariate linear regression was used to analyse the relationships between the biomass of sheep and cattle per farm and ABU.

## Results

**Figure 1.**
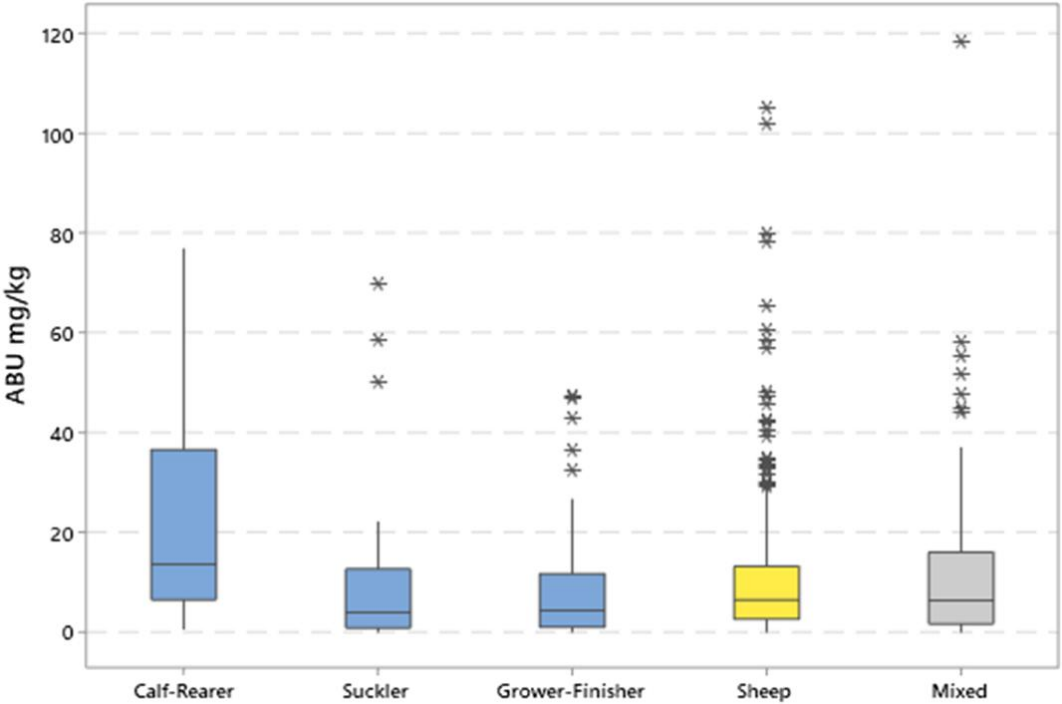
ABU distributions in mg/kg (UK) for individual farms in each production system category. Outliers farms that are at least 1.5 times the IQR are identified by an asterisk (*), in addition to the median line and IQR box. This figure appears in colour in the online version of *JAC* and in black and white in the print version of *JAC*.

**Figure 2.**
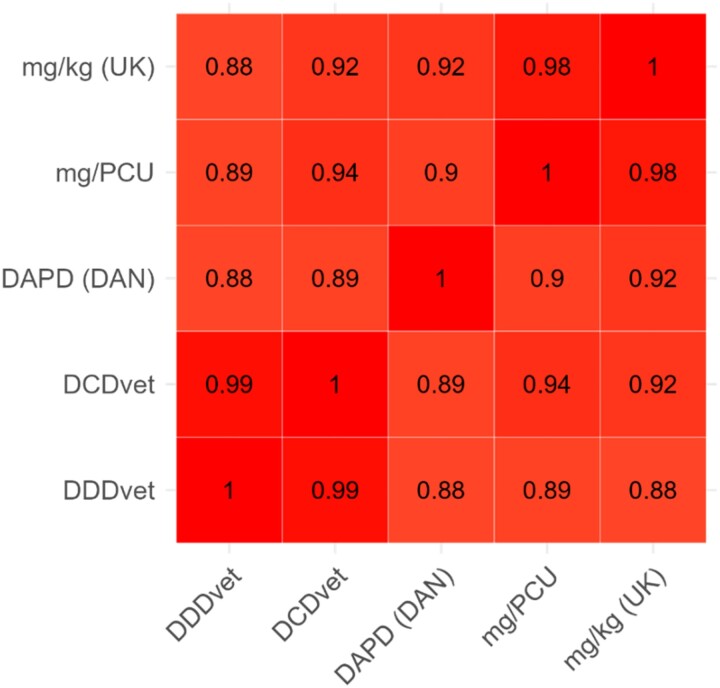
Correlations between metrics for sheep farms. DAN, Denmark. This figure appears in colour in the online version of *JAC* and in black and white in the print version of *JAC*.

**Figure 3.**
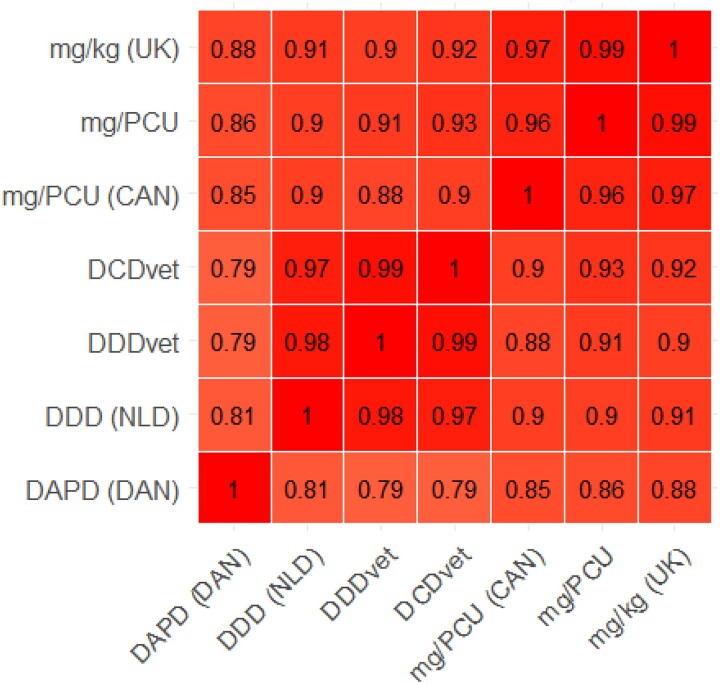
Correlations between metrics for beef cattle farms. CAN, Canada; NLD, The Netherlands; DAN, Denmark. This figure appears in colour in the online version of *JAC* and in black and white in the print version of *JAC*.

Antibiotic usage is shown in Table 1 by each metric for farms in each of the seven farm categories. All categories show the same highly skewed distribution, with a small proportion of ‘high-user’ outlier farms substantially increasing the mean ABU relative to the median ABU value for each population, as shown in Figure 1 for mg/kg (UK). The proportional distribution of ABU by antibiotic class is described for the mg/kg (UK) metric in Table 2. Correlation between ABU metrics was generally high. A matrix for farms with sheep and no beef cattle is shown in Figure 2, with correlations for beef cattle farms with no sheep shown in Figure 3.

**Table 2.** Summary of ABU as percent contribution of each active ingredient class in total mg/kg for each of the production systems. The dose rate (mg/kg body weight) varies substantially according to class and individual antibiotic product

*Antibiotic class*	*Sheep* and c*alf*-r*earer* (%)	*Sheep* and g*rower-*f*inisher* (%)	*Sheep* and s*uckler* (%)	*Calf-*r*earer* (%)	*Grower-*f*inisher* (%)	*Suckler* (%)	*Sheep* (%)
*Aminoglycosides*	28.6	20.3	19.3	11.2	19.5	26.2	21.1
β-L*actams*	25.8	25.0	33.0	26.2	30.8	29.8	19.3
*Fluoroquinolones*	0.1	0.1	0.0	0.1	0.1	0.0	0.0
*Macrolides*	14.4	5.4	9.8	8.9	6.3	13.4	3.3
*Trimethoprim/sulphonamide*	5.4	3.0	0.3	5.1	3.7	4.1	1.9
*Tetracyclines*	24.8	40.0	37.2	33.4	33.9	25.5	49.4
*Other classes*	1.1	6.2	0.4	15.2	5.7	0.9	5.1

**Figure 4.**
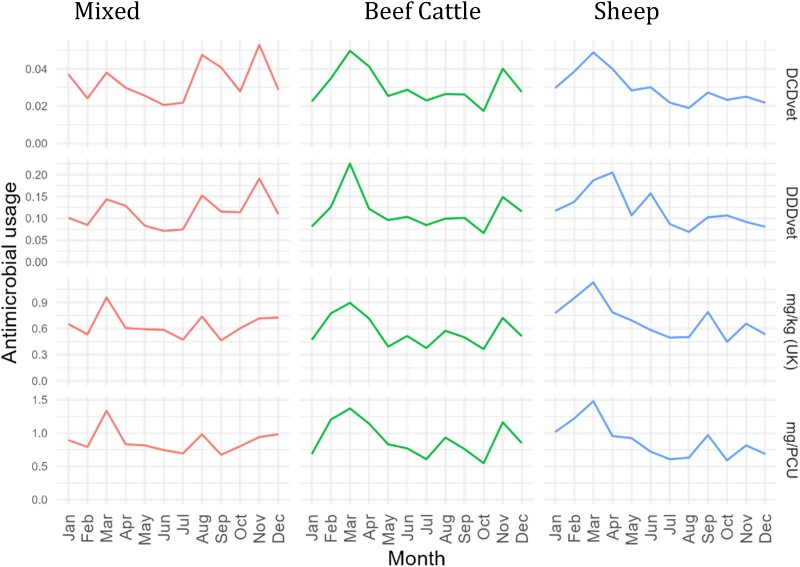
Seasonality of ABU by a variety of metrics for beef cattle, sheep and mixed cattle and sheep farms (note *y*-axis scales differ). This figure appears in colour in the online version of *JAC* and in black and white in the print version of *JAC*.

Strong seasonal patterns were observed in ABU for all groups with higher use in winter and spring, coinciding with winter housing of cattle and lambing, respectively (Figure 4).

**Figure 5.**
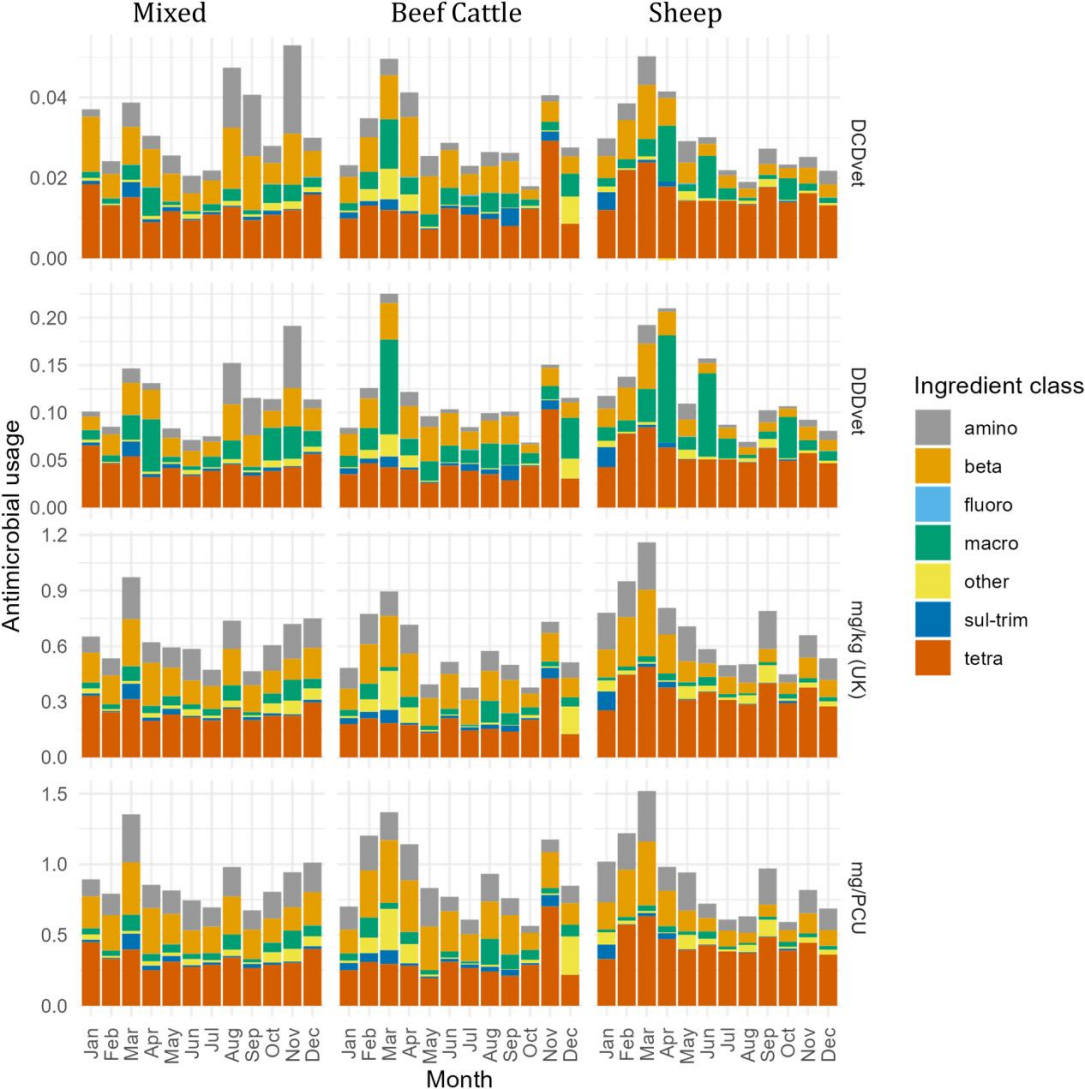
ABU by class and month for beef cattle, sheep and mixed beef cattle and sheep farms by a variety of metrics (note *y*-axis scales differ). Amino, aminoglycosides; beta, β-lactams; fluoro, fluoroquinolones; macro, macrolides; sul-trim, trimethoprim/sulphonamide; tetra, tetracyclines. This figure appears in colour in the online version of *JAC* and in black and white in the print version of *JAC*.

Antibiotic use in both beef cattle and sheep was dominated by tetracycline and β-lactams, (Figure 5). However, seasonal patterns in beef cattle systems were disproportionately driven by use of the macrolide antibiotics tylosin, gamthithromycin and tulathromycin (Appendix iv).

### Beef farm type

**Figure 6.**
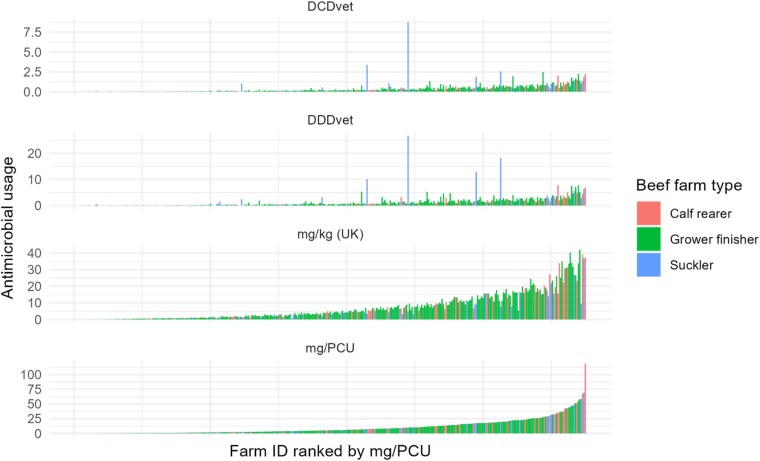
Distribution of individual farm ABU for the three exclusively beef cattle farm management system types (calf-rearer, red; grower finisher, green; suckler, blue), by the UK metric [mg/kg (UK)] and the EU metrics (DCDvet, DDDvet and mg/PCU). This figure appears in colour in the online version of *JAC* and in black and white in the print version of *JAC*.

The distribution of farm-level ABU rankings from highest to lowest for all the beef herds from the three different production systems (beef cattle only) was compared across the principal UK and EU dose- and mass-based metrics (Figure 6). Agreement between metrics was highest between the EU and UK mass-based metrics. Agreement was lower between mass and dose metrics, with poor agreement between metrics for suckler herds in particular.

**Figure 7.**
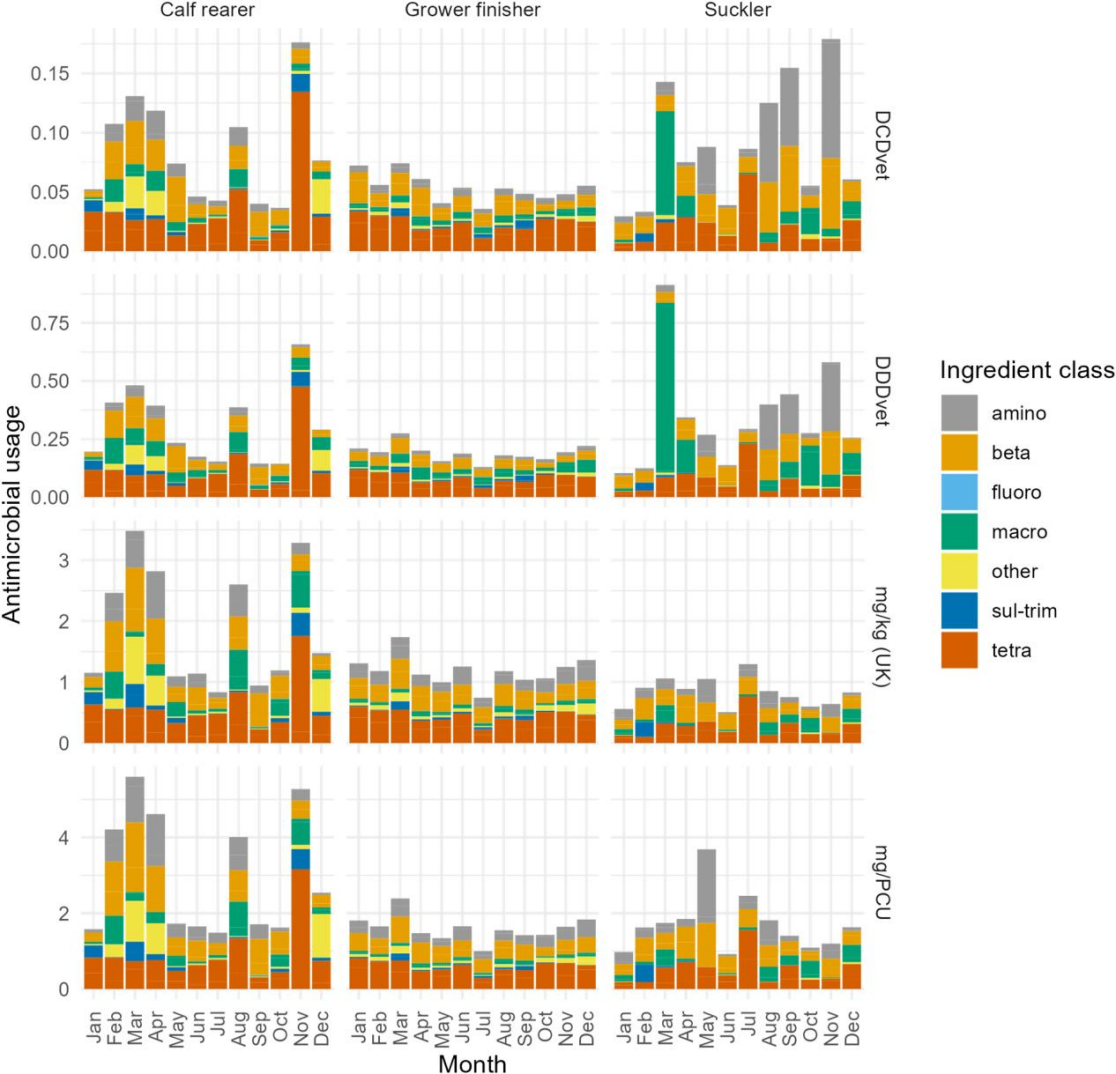
ABU by month and class for beef farm subtypes. Amino, aminoglycosides; beta, β-lactams; fluoro, fluoroquinolones; macro, macrolides; sul-trim, trimethoprim/sulphonamide; tetra, tetracyclines. This figure appears in colour in the online version of *JAC* and in black and white in the print version of *JAC*.

ABU ranking by mg/kg (UK) was analysed by beef farm subtype (Figure 7), identifying significantly higher use among calf-rearers compared with other farm types and more volatility over time in both absolute ABU and relative ABU by antibiotic class.

The overall farm size (PCU on the farm of both cattle and sheep) and the farm composition (proportion of biomass cattle versus sheep) was assessed against mg/PCU (EU). Univariate linear regression analysis suggested an association between proportion of cattle (in kg biomass) and mg/PCU (EU), with a 1% increase in cattle biomass being equivalent to a 0.04 (95% CI 0.02–0.07) mg/PCU increase in ABU. A negative association was observed with univariate linear regression analysis of total PCU (kg) on the farm and ABU, with each 1000 kg being associated with a −0.01 mg (95% CI −0.02 to 0.00), equating to an increase in farm size of 100 cows or approximately 570 sheep (i.e. ∼42 500 kg) being associated with a 0.425 mg/PCU (EU) decrease in ABU. There was a relatively small absolute difference in mean ABU between farms with 0%–25% of cattle [7.38 mg/PCU (EU)], 26%–50% of cattle [8.40 mg/PCU(EU)], 51%–75% of cattle [8.92 mg/PCU(EU)] or 76%–100% of cattle [11.14 mg/PCU(EU)].

## Discussion

This study quantifies and compares ABU patterns in different beef cattle and mixed farming systems for the first time, to our knowledge, on a large national scale using veterinary prescribing records. Farm-level ABU in both beef cattle, sheep and mixed farms was relatively low when compared with other livestock species by all the metrics tested. In spite of the fact that some of the metrics (mg/PCU, DDDvet and DCDvet) were designed for national rather than farm-level monitoring, this study demonstrates that the ranking of farms in terms of ABU level is relatively consistent. Whilst the individual figures do vary between the ABU metrics, the potential to identify ‘high ABU’ farms is relatively similar. Where substantial variation was observed between metrics seasonally and at the level of individual farms, this was driven by use of a small number of specific antibiotic classes such as long-acting macrolides and by specific antibiotic administration/formulations, principally intramammary tubes.

In the UK and EU, there has been considerable interest in linking health, welfare and ABU to direct farm subsidy payments in a variety of ways. This could be used as both an incentive to change behaviour and agricultural practice, or as a component of a risk-based surveillance system for ABR in the environment or food chain, arising as a consequence of ABU in agriculture. To achieve this, a common metric for ABU needs to be used to benchmark farms, and several have been developed in Europe and North America for this purpose. Farmers and policymakers need to have confidence that the metrics used to benchmark ABU at the farm level are fair, accurate and robust and our study has sought to examine how real farm data would be ranked by different ABU metrics. Our study indicates that most of the metrics demonstrate good agreement, with some caveats. The mg/kg PCU (EU) metric tends to produce a higher figure than the mg/kg (UK) for beef. This is because the mg/kg (UK) for beef takes into account all beef cattle on the farm and uses weights based on ‘average liveweight’, whereas the mg/PCU only incorporates the number of slaughtered beef cattle and uses weight based on ‘average weight at time of treatment’ (which is lower than the liveweight). The mg/kg PCU (Canada) also produces a lower figure for cattle than the ESVAC mg/kg PCU (EU). This is because, although the denominator weights are based on ‘average weight at time of treatment’, breeding beef cattle are also included alongside slaughter cattle and the weights are higher for breeding animals than slaughtered beef animals (adapted to the Canadian context). The mg/kg PCU (EU) for sheep, by contrast to beef, also includes breeding sheep, which helps explain why the difference between sheep only and cattle only farms is greater for mg/PCU than mg/kg. When looking at the course dose metrics, the DAPD (Denmark) metric tends to produce a higher figure than the ESVAC DDDvet (EU) figure, largely because they relate to the average number of doses per 1000 animals per day (i.e. 1000 animal days) as opposed to per calendar year for ESVAC, which relates to the average number of doses per animal per year (i.e. 365 animal days).

The overarching purpose of any farm-level benchmarking system is to identify the farms that pose the greatest ABR risk. In this study we can only use ABU as a proxy and this excludes many other factors that are likely to influence ABR, such as diet.^3^ However, the demographic and ABU data presented here do at least allow us to identify the most obvious group that could be classified as a ‘high risk’ of being a high antibiotic user. These are ‘calf-rearer’ farms rearing dairy and dairy/beef cross calves on artificial milk replacer systems, where those animals are destined for beef production. These systems commonly manage larger numbers of young animals at a high stocking density, which increases the risk of infectious diseases that require antibiotic treatment. Increased ABU in calf-rearing populations is also likely due to mixing of calves from multiple farms, which have often passed through sorting centres. Young calves are represented in three of the production system types characterized in this study (calf-rearers, suckler, mixed breeding sheep and calf-rearer). It is not possible from the data available to compare the ABU administered to calves in the same age range across the three production systems. However, it may be reasonable to assume that the more extensive nature of spring calving suckler herds reduces the risk of disease and antibiotic treatment. Other farm management factors, which are likely to be multifactorial, go beyond the scope of this study. In contrast to the sucker versus calf-rearer comparison we have a different issue with the high biomass and typically low ABU of breeding sheep relative to young calves in the ‘mixed breeding sheep/calf-rearer’ category. This combination of factors obscures the true ABU that may be occurring in the calves on these farms. This is an inherent problem when using unhypothecated recording of usage/antibiotic sales for usage surveillance and benchmarking in a population of farms that operate a wide variety of management systems across multiple species.

In several instances, specific metrics reflect a more realistic distribution of true ABU than others, for example the UK metrics are more suited to beef farms that don’t produce slaughter animals because they incorporate more weight bands for growing cattle. The importance of this becomes very clear when metrics developed for national surveillance are used by various parties to compare usage between production systems in a way they were never intended for. If governmental or industry stakeholders wish to benchmark farms more accurately and fairly it will become increasingly important to develop a metric that incorporates the weight change of the animals over their time on-farm as the denominator. This is possible and feasible in the UK context where we have pre-existing, centralized, traceability systems to record births and deaths as well as track movements of animals between farms using individual animal identifiers linked to key information such as age and breed (in the case of cattle). This information would be sufficient to develop a ‘*kg of livestock at risk of treatment*’ denominator for an ABU metric.

Decisions taken by policymakers on the control and surveillance of ABU need to use robust, comprehensive data streams. Antibiotic sales/prescribing data is the only practical data type to achieve this as other methodologies such as voluntary reporting of usage or bin surveys are inherently far more labour-intensive for farmers and are self-evidently more difficult to automate and audit. They are inherently more prone to systematic underreporting and underestimation of ABU where as sales-based surveillance can be automated and provide an upper estimate of usage rather than an underestimate. This study describes how several metrics perform similarly and provides some confidence that within a given year the choice of metric correctly classifies a high proportion of farms. This is important when the outcome of a classification based on the ABU metric may be used as part of a subsidy payment framework, as has been suggested in the UK.

## Supplementary Material

dkad259_Supplementary_Data

## Supplementary data

Appendices i to iv are available as Supplementary data at *JAC* Online.
