# Supplementary material for: Comparison of multiple international metrics for benchmarking antibiotic usage (ABU) using UK beef and sheep data
Source: J Antimicrob Chemother. 2023 Aug 16;78(10):2496–504. doi: 10.1093/jac/dkad259 (PMC10545515; doi:10.1093/jac/dkad259)
Supplement: dkad259_Supplementary_Data [file dkad259_supplementary_data.docx]

**Appendix i**

**Summary of numerator and denominator categories used for ABU metrics.**

| Metric | Numerator | Bovine denominator | Ovine denominator | Source | Notes |
| --- | --- | --- | --- | --- | --- |
| mg/PCU | mg | Live dairy cattle (425kg), slaughtered cattle (425kg), slaughtered calves (140kg) | Ewe numbers (75kg), slaughtered sheep (20kg) not including store lambs | (ESVAC, 2020) | |
| DDDvet | mg/dose for parenteral/oral products, doses/animal for lactational intramammary and intrauterine tubes | Live dairy cattle (425kg), slaughtered cattle (425kg), slaughtered calves (140kg),  Number for lct and iu tubes | Ewes (75kg), slaughtered sheep (20kg) | (ESVAC, 2020) | |
| DCDvet | mg/course for parenteral/oral products, courses/animal for lactational & dry period intramammary and intrauterine tubes | Live dairy cattle (425kg), slaughtered cattle (425kg), slaughtered calves (140kg),  Number for lct, iu and dct tubes | Ewes (75kg), slaughtered sheep (20kg) | (ESVAC, 2020) | |
| mg/kg (UK) | mg | Adult female (670kg), adult male (760kg), 18-24mo (584kg), 12-24mo (507.5kg), <12mo (193.25kg) | Ewes (75kg), store lambs and lambs (20kg) | (CHAWG, 2020) | Where sex unknown a mean of male/female weight was taken |
| DDD (NLD) | mg/dose | Adult female (600kg), adult male (800kg), 18-24mo (628kg), 12-24mo (628kg), <12mo (283kg) | None | (SDa, 2019) | Dose values from ESVAC utilised as values from dg-standard was not available |
| DAPD (DAN) | mg/dose | Adult female (600kg), adult male (600kg), 18-24mo (300kg), 12-24mo (300kg), <12mo (100kg) | Ewes (50kg), store lambs and lambs (20kg) | (DANMAP, 2019) | |
| mg/PCU (CAN) | mg | Live dairy cattle (425kg), live beef cattle (425kg), slaughtered cattle (425kg), slaughtered calves (140kg) | None | (CIPARS, 2015) | |

**Appendix ii - Monthly analysis of ABU (mg/PCU) for cattle, sheep and mixed farms for**

**antibiotic classes**

| Species | Month | amino | beta | fluoro | macro | other | sul-trim | tetra |
| --- | --- | --- | --- | --- | --- | --- | --- | --- |
| Mixed | Jan | 0.12 | 0.29 | 0 | 0.04 | 0.04 | 0.02 | 0.45 |
| Mixed | Feb | 0.15 | 0.25 | 0 | 0.04 | 0.01 | 0.01 | 0.33 |
| Mixed | Mar | 0.34 | 0.37 | 0 | 0.11 | 0.02 | 0.11 | 0.4 |
| Mixed | Apr | 0.17 | 0.33 | 0 | 0.05 | 0.03 | 0.03 | 0.25 |
| Mixed | May | 0.17 | 0.22 | 0 | 0.06 | 0.02 | 0.04 | 0.31 |
| Mixed | Jun | 0.21 | 0.17 | 0 | 0.04 | 0.04 | 0.01 | 0.27 |
| Mixed | Jul | 0.14 | 0.19 | 0 | 0.05 | 0.02 | 0.02 | 0.29 |
| Mixed | Aug | 0.23 | 0.28 | 0 | 0.11 | 0.04 | 0.01 | 0.34 |
| Mixed | Sep | 0.15 | 0.22 | 0 | 0.02 | 0.01 | 0.03 | 0.27 |
| Mixed | Oct | 0.19 | 0.17 | 0 | 0.07 | 0.07 | 0.02 | 0.29 |
| Mixed | Nov | 0.26 | 0.17 | 0 | 0.13 | 0.09 | 0.01 | 0.3 |
| Mixed | Dec | 0.21 | 0.23 | 0 | 0.08 | 0.07 | 0.02 | 0.4 |
| Pure cattle | Jan | 0.16 | 0.17 | 0 | 0.05 | 0.02 | 0.05 | 0.25 |
| Pure cattle | Feb | 0.25 | 0.33 | 0 | 0.14 | 0.11 | 0.06 | 0.31 |
| Pure cattle | Mar | 0.2 | 0.44 | 0 | 0.04 | 0.29 | 0.1 | 0.29 |
| Pure cattle | Apr | 0.26 | 0.36 | 0 | 0.08 | 0.14 | 0.02 | 0.28 |
| Pure cattle | May | 0.27 | 0.31 | 0 | 0.03 | 0.01 | 0.01 | 0.2 |
| Pure cattle | Jun | 0.1 | 0.28 | 0 | 0.04 | 0.01 | 0.02 | 0.31 |
| Pure cattle | Jul | 0.1 | 0.19 | 0 | 0.01 | 0.01 | 0.02 | 0.27 |
| Pure cattle | Aug | 0.2 | 0.26 | 0 | 0.18 | 0.01 | 0.03 | 0.24 |
| Pure cattle | Sep | 0.12 | 0.28 | 0 | 0.1 | 0 | 0.04 | 0.21 |
| Pure cattle | Oct | 0.05 | 0.12 | 0 | 0.07 | 0.02 | 0.01 | 0.29 |
| Pure cattle | Nov | 0.09 | 0.25 | 0 | 0.04 | 0.01 | 0.08 | 0.7 |
| Pure cattle | Dec | 0.12 | 0.15 | 0 | 0.08 | 0.27 | 0 | 0.22 |
| Pure sheep | Jan | 0.17 | 0.11 | 0 | 0.01 | 0.05 | 0.06 | 0.19 |
| Pure sheep | Feb | 0.16 | 0.2 | 0 | 0.02 | 0.01 | 0 | 0.33 |
| Pure sheep | Mar | 0.21 | 0.26 | 0 | 0.02 | 0.01 | 0.01 | 0.36 |
| Pure sheep | Apr | 0.09 | 0.14 | 0 | 0.02 | 0 | 0.02 | 0.27 |
| Pure sheep | May | 0.16 | 0.08 | 0 | 0.01 | 0.06 | 0 | 0.23 |
| Pure sheep | Jun | 0.06 | 0.05 | 0 | 0.03 | 0.02 | 0 | 0.25 |
| Pure sheep | Jul | 0.05 | 0.07 | 0 | 0.01 | 0.01 | 0 | 0.22 |
| Pure sheep | Aug | 0.07 | 0.04 | 0 | 0.01 | 0.03 | 0 | 0.21 |
| Pure sheep | Sep | 0.15 | 0.05 | 0 | 0.01 | 0.07 | 0 | 0.28 |
| Pure sheep | Oct | 0.03 | 0.05 | 0 | 0.02 | 0.01 | 0.01 | 0.23 |
| Pure sheep | Nov | 0.1 | 0.08 | 0 | 0.02 | 0.02 | 0 | 0.26 |
| Pure sheep | Dec | 0.09 | 0.06 | 0 | 0.02 | 0.02 | 0 | 0.21 |

**Appendix iii - Farm demographic data collection questionnaire**

**Complete all sections that apply**

Organic/conventional

**Suckler herd**

Number of breeding cows: _______________

Proportion of calf crop sold: 1) As weaned calves < 1 year old: ___________

2) As stores > 1 year old: ________

3) Finished: ___________ Target age for finished cattle: ________

4) Retained for breeding: ____________

Number of purchased replacement heifers: _____________

**Rearing**

Number of dairy calves **on milk** purchased/year: __________

Proportion of purchased calves sold: 1) As weaned calves < 1 year old: ___________

2) As stores > 1 year old: ________

3) Finished: _______ Target age for finished cattle: _______

**Growing and finishing**

Weaned calves < 1 year old purchased/year: ___________

Stores > 1 year old purchased/year: ____________

Proportion of purchased calves sold: 1) For finishing: __________

2) Finished: _______ Target age for finished cattle: _______

**Sheep**

Number of breeding ewes >2 years old: _______________

Number of breeding ewes <2 years old: _______________

Hill/upland/lowland: ______________

**Appendix iv**


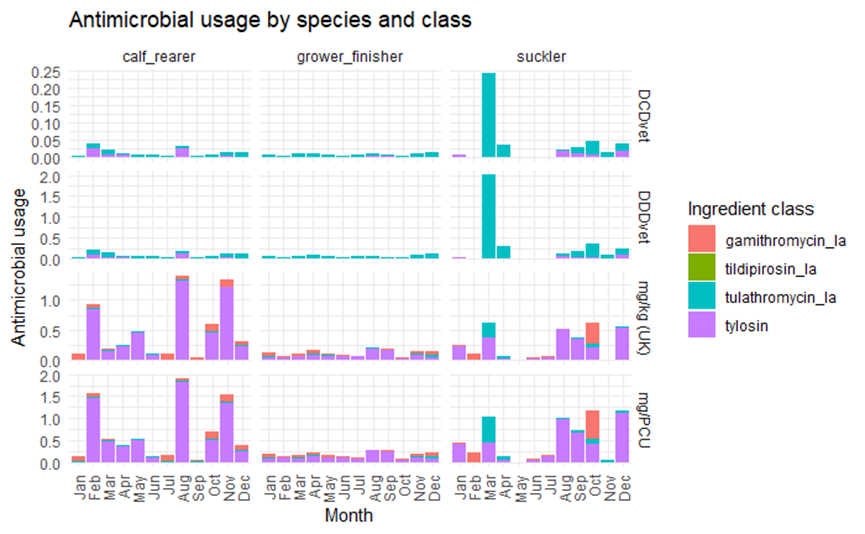


Figure S1. Macrolide antibiotic use in beef farm systems by month and ABU indicator metric.
